# *In Vivo* SPECT Reporter Gene Imaging of Regulatory T Cells

**DOI:** 10.1371/journal.pone.0025857

**Published:** 2011-10-17

**Authors:** Ehsan Sharif-Paghaleh, Kavitha Sunassee, Richard Tavaré, Kulachelvy Ratnasothy, Alexander Koers, Niwa Ali, Rowa Alhabbab, Philip J. Blower, Robert I. Lechler, Lesley A. Smyth, Gregory E. Mullen, Giovanna Lombardi

**Affiliations:** 1 Medical Research Council (MRC) Centre for Transplantation, King's College London, King's Health Partners, Guy's Hospital, London, United Kingdom; 2 Division of Imaging Sciences, King's College London, St Thomas Hospital, London, United Kingdom; Centre de Recherche Public de la Santé (CRP-Santé), Luxembourg

## Abstract

Regulatory T cells (Tregs) were identified several years ago and are key in controlling autoimmune diseases and limiting immune responses to foreign antigens, including alloantigens. *In vivo* imaging techniques including intravital microscopy as well as whole body imaging using bioluminescence probes have contributed to the understanding of *in vivo* Treg function, their mechanisms of action and target cells. Imaging of the human sodium/iodide symporter via Single Photon Emission Computed Tomography (SPECT) has been used to image various cell types *in vivo*. It has several advantages over the aforementioned imaging techniques including high sensitivity, it allows non-invasive whole body studies of viable cell migration and localisation of cells over time and lastly it may offer the possibility to be translated to the clinic. This study addresses whether SPECT/CT imaging can be used to visualise the migratory pattern of Tregs *in vivo*. Treg lines derived from CD4^+^CD25^+^FoxP3^+^ cells were retrovirally transduced with a construct encoding for the human Sodium Iodide Symporter (NIS) and the fluorescent protein mCherry and stimulated with autologous DCs. NIS expressing self-specific Tregs were specifically radiolabelled *in vitro* with Technetium-99m pertechnetate (^99m^TcO_4_^−^) and exposure of these cells to radioactivity did not affect cell viability, phenotype or function. In addition adoptively transferred Treg-NIS cells were imaged *in vivo* in C57BL/6 (BL/6) mice by SPECT/CT using ^99m^TcO_4_^−^. After 24 hours NIS expressing Tregs were observed in the spleen and their localisation was further confirmed by organ biodistribution studies and flow cytometry analysis. The data presented here suggests that SPECT/CT imaging can be utilised in preclinical imaging studies of adoptively transferred Tregs without affecting Treg function and viability thereby allowing longitudinal studies within disease models.

## Introduction

Regulatory T cells (Tregs) are key players in the adaptive immune system. Tregs are responsible for maintaining immune homeostasis and self-tolerance by preventing autoimmunity and limiting immune responses to foreign antigens such as alloantigens [1]. The regulatory function of Tregs on CD4^+^ or CD8^+^ effector T cells (Teff) has made them a potential immunotherapy agent for clinical treatments of various diseases [2]. Applications of Tregs in the field of transplantation has been recognised as adjunct therapy to immunosuppressive drugs to induce transplantation tolerance [3]. Adoptive transfer of murine derived Tregs has been a successful strategy in the prevention of graft rejection and in the cure of autoimmune diseases such as diabetes. More recently injection of human Tregs into humanised mouse models lead to the protection from vessel and skin transplant pathology [4], [5]. Furthermore, injection of *ex-vivo* expanded Tregs has been used in clinical trials to prevent Graft versus Host Disease (GvHD) in bone marrow transplant patients [6].

However, to date the mechanisms by which adoptively transferred Treg function *in vivo* and where they exert their suppressive function remains unclear. Imaging techniques have been utilised to gain some understanding of their mechanisms of action and location. Intravital two-photon laser scanning microscopy was utilised by Tang et al (2006) to demonstrate that suppression of T cells was a consequence of the interaction between Tregs and DCs [7]. In another study, Zhang et al (2009) utilised immunofluorescent imaging of adoptively transferred Tregs in an islet transplant model and observed that Treg sequential migration from blood to tissue and then to the draining lymph nodes was required for their optimal suppressive capacity *in vivo* [8]. In another study, fluorescent imaging was utilised to demonstrate that the aforementioned sequential migration of Tregs was necessary for optimal suppression during a skin immune response in the form of contact hypersensitivity [9]. Although these studies have helped in understanding Treg function *in vivo* there are some limitations associated with the imaging techniques utilised. The major drawback in using intravital microscopy as an initial imaging technique is that the location of the cells of interest needs to be known (which is predominately limited by the technique to the skin or lymph nodes) and the optimal time to image. The major advantage of whole body imaging using radioisotopes is that it is a fully quantitative technique and one is not biased or limited by the “expected” biodistribution of the cells of interest.

Imaging modalities such as Positron Emission Tomography (PET) and Single Photon Emission Computed Tomography (SPECT) are routine clinical imaging techniques used within Nuclear Medicine and have been utilised to image various cells including T cells [10], [11], [12], [13]. Cells visualised with these techniques can be labelled directly or via reporter gene mediated uptake of radioisotopes [13]. Although direct radiolabelling of cells can be a rapid procedure, it has the disadvantage of being short lived, not linked to cell viability and not necessarily imaging of the labelled cells of interest (i.e. labelled cells undergo cell death and the radiolabel passes to phagocytic cells) [13]. In contrast, receptor mediated radiolabelling does not have these limitations and has the potential to image viable cells longitudinally. The expression of the reporter gene human Sodium Iodide Symporter (NIS) and the incorporation of isotopes such as Technetium-99m pertechnetate (^99m^TcO_4_^−^), Rhenium-188 perrhenate and Iodine through NIS has been utilised to label and image various cells such as hepatocarcinoma cells, cervix tumour cells, glioma cells and mammary gland cells [14], [15], [16], [17], [18].

In this study, we investigated whether Tregs can be transduced to express NIS and whether NIS expressing Tregs maintained their phenotype and functions when radiolabelled *in vitro* and finally whether they could be imaged *in vivo* using NanoSPECT/CT. The results from this study suggest that SPECT technology is a useful tool for tracking Tregs *in vivo* longitudinally and to help in understanding Treg migration, distribution, mechanism of action and life span in various disease models.

## Materials and Methods

### Ethics statement

Animal studies were carried out in accordance with UK Research Councils' and Medical Research Charities' guidelines on Responsibility in the Use of Animals in Bioscience Research, under a UK Home Office license (PPL 70/6473; Title: Mechanisms of immunological response to foreign antigens).

### Mice, culture media, reagents, antibodies and Flow cytometric analysis

C57BL/6 (BL/6) (H-2^b^) mice aged 6–8 weeks old were purchased from Harlan. RPMI 1640 medium supplemented with L-glutamine (Gibco, Invitrogen), penicillin/streptomycin (Gibco, Invitrogen), and 10% (v/v) FCS (PAA Laboratories) was used for all *in vitro* assays. Anti-CD4–FITC (clone GK1.4), anti-FoxP3–APC (clone FJK-16s) and anti-CD25–PE (clone PC61) were purchased from eBioscience. All flow cytometry analysis was conducted on a BD FACS LSR II using FACs DIVA software (BD Biosciences). For surface staining, 5×10^5^ cells were incubated with saturating concentrations of appropriate antibodies for 30 minutes at 4°C, then washed twice in cold FACS buffer (PBS with 1% [v/v] FCS) before analysis. For intracellular staining of FoxP3, a mouse FoxP3 staining kit was used (eBioscience) according to manufacturer's protocol.

### Cell preparation

*Tregs:* Treg lines were generated as described earlier [19]. Briefly, CD4^+^CD25^+^ Tregs were isolated from the lymph nodes and spleen of BL/6 mice using a CD4^+^CD25^+^ selection kit (Miltenyi Biotec). For generation of Treg lines, self-specific CD4^+^CD25^+^ Tregs (2×10^6^ cells/mL) were stimulated once per week with immature BL/6 DCs (0.5×10^6^/mL) in the presence of 10 U/mL of IL-2 in a 24-well plate.

*DC:* Bone marrow derived DCs (BM-DCs) were generated as described earlier [20] according to the protocol described by Inaba and colleagues [21]. Briefly, the bone marrow was treated with ACK lysing buffer and incubated for 30 minutes at 4°C with anti-CD4, -CD8, -B220 and anti-MHC Class II supernatants (YTS169, YTS191, RA34.5, M5/114 respectively). Cells were washed with RPMI and Dynal beads (Gibco, Invitrogen) coated with goat anti-rat IgG were then added. Beads and cells were incubated for 30 minutes at 4°C before being placed on a magnet. DCs were then cultured at 37°C/5% CO_2_ using FCS media supplemented with mouse GM-CSF. Media was changed on days 2 and 4 to remove non-adherent cells. DCs were used on day 6 of culture.

### Production of NIS vector, retroviral supernatant and retroviral transduction

Retroviral supernatant was produced as previously described [19] with the following modification. The MSCV (Murine Stem Cell Virus) MIGR1 vector [22] was modified to contain human NIS followed by the IRES element and mCherry. NIS in the pcDNA3 vector [23] was amplified by PCR using the forward primer 5′-CAC CAC CTC GAG GCT GTC AGC GCT GAG CAC AGC and reverse primer 5′-CAC CAC GAA TTC TTT GGC CCA TCC TGA GGA ACC. The resulting PCR product was digested with the restriction enzymes XhoI and EcoRI (NEB) and ligated into the XhoI and EcoRI digested MIGR1 vector. Next, mCherry obtained from the lab of Professor R.Y. Tsien [24] was amplified by PCR using the forward primer 5′-CAC CAC GGT CTC CCA TGC GCC ACC ATG GTG AGC AAG G and reverse primer 5′- CAC CAC GTC GAC CTT GTA CAG CTC GTC CAT GCC. This PCR product was digested with Esp3I, leaving an NcoI-compatible overhang, and SalI, and subsequently ligated into the NIS containing MIGR1 vector that had been digested with NcoI and SalI, which had removed the GFP. The resulting pNImC vector was analyzed for the correct sequence and then transfected into Phoenix-Eco packaging cells using CaPO_4_. Supernatant was collected on day 2. For retroviral transduction, Tregs were first activated with autologous DCs and anti-CD3ε (1 µg/mL) for 48 hours. The activated cells were then transferred to a RetroNectin-coated (Takara) 24-well plate (2–3×10^6^/mL in single well of 24-well plate) with 50% (v/v) retroviral supernatant supplemented with 10 U/mL of IL-2 and cultured at 37°C for 24 hours. Supernatants were removed, and the cells cultured in fresh medium supplemented with 10 U/mL of IL-2. The NIS transduced self-specific Tregs (B6s-NIS) were enriched by flow cytometry cell sorting (BD FACS Aria II cell sorter) by gating on the mCherry positive cells.

#### Treg Suppression Assay

Tregs (50 µl of 1×10^6^ cells/mL) were co-cultured with CD4^+^ T cells (50 µl of 1×10^6^ cells/mL) isolated from BL/6 mice in the presence of T cell–depleted splenocytes generated from BL/6 mice (5o µl of 2×10^6^ cells/mL) and anti-CD3ε (1 µg/mL) in a 96-well plate. Proliferation was assessed by measuring [^3^H] thymidine incorporation during the last 18 hours of a 3-day culture.

### *In vitro* radiolabelling and cell viability assays

For assessing receptor mediated ^99m^TcO_4_^−^ uptake *in vitro*, transduced (B6s-NIS) and non-transduced (B6s) Tregs were washed in Hank's Balanced Salt solution (Gibco, Invitrogen) before 1×10^6^ Tregs were incubated with 1 MBq of ^99m^TcO_4_^−^ (kindly provided by Dept. of Nuclear Medicine, Guy's and St. Thomas' Hospital) for 15 minutes at 37°C. Cells were then pelleted and the supernatant carefully removed. The amount of ^99m^TcO_4_^−^ uptake was assessed using a Compugamma 1281 gamma counter (LKB Wallac). Included in same experiment was the NIS inhibitor, Sodium Perchlorate (100 µM per 1×10^6^ cells). The toxicity effect of radioactivity on B6s-NIS Tregs was measured using a standard MTT assay (Molecular Probes, Invitrogen). B6s-NIS Tregs (1×10^6^) were incubated with 0–100 MBq of ^99m^TcO_4_^−^ for 15 minutes at 37°C. Cells were suspended in FCS containing media without Phenol red and 1×10^5^ B6s-NIS Tregs were then plated into a 96-well plate in triplicates and a MTT assay was carried out according to manufacturer's protocol. Plates were read at a wavelength of 570 nm using a Bio-Tek EL800 automatic plate reader (Wolf laboratories).

Also 1×10^6^ B6s-NIS Tregs cells were radiolabelled with 30 MBq of ^99m^TcO_4_^−^ and suppression assay performed as described above.

### Adoptive transfer and NanoSPECT/CT imaging of Tregs in vivo

BL/6 mice were injected intravenously (i.v) with 1×10^6^ Carboxyfluorescein succinimidyl ester (CFSE) labelled (1 µM, Molecular Probes, Invitrogen) B6s-NIS Tregs 24 hours before imaging. On the day of scanning, 20 MBq of ^99m^TcO_4_^−^ was administered i.v and the mouse was imaged, under inhaled isofluorane anaesthetics for 1 hour using a NanoSPECT/CT preclinical imager (Bioscan) equipped with a multipinhole (nine pinholes, aperture 1.0 mm) collimator. Images were then reconstructed using the Invivoscope software (Bioscan). Mice were then culled and ^99m^TcO_4_^−^ biodistribution studies and FACS analysis were performed at the end of the scanning. Standard uptake values of ^99m^TcO_4_^−^ were calculated by the formula: [CPM (organ)/weight (organ)]/[CPM (whole mouse)/weight (whole mouse)].

### Statistics

Data are expressed as mean of triplicates ± SEM. Significance between 2 groups (B6s and B6s-NIS Tregs) was calculated using unpaired 2-tailed t test using GraphPad Prism 5 software. P values less than 0.05 were considered statistically significant.

## Results

### Tregs transduced with NIS maintain their phenotypic and suppressive function

**Figure 1:**
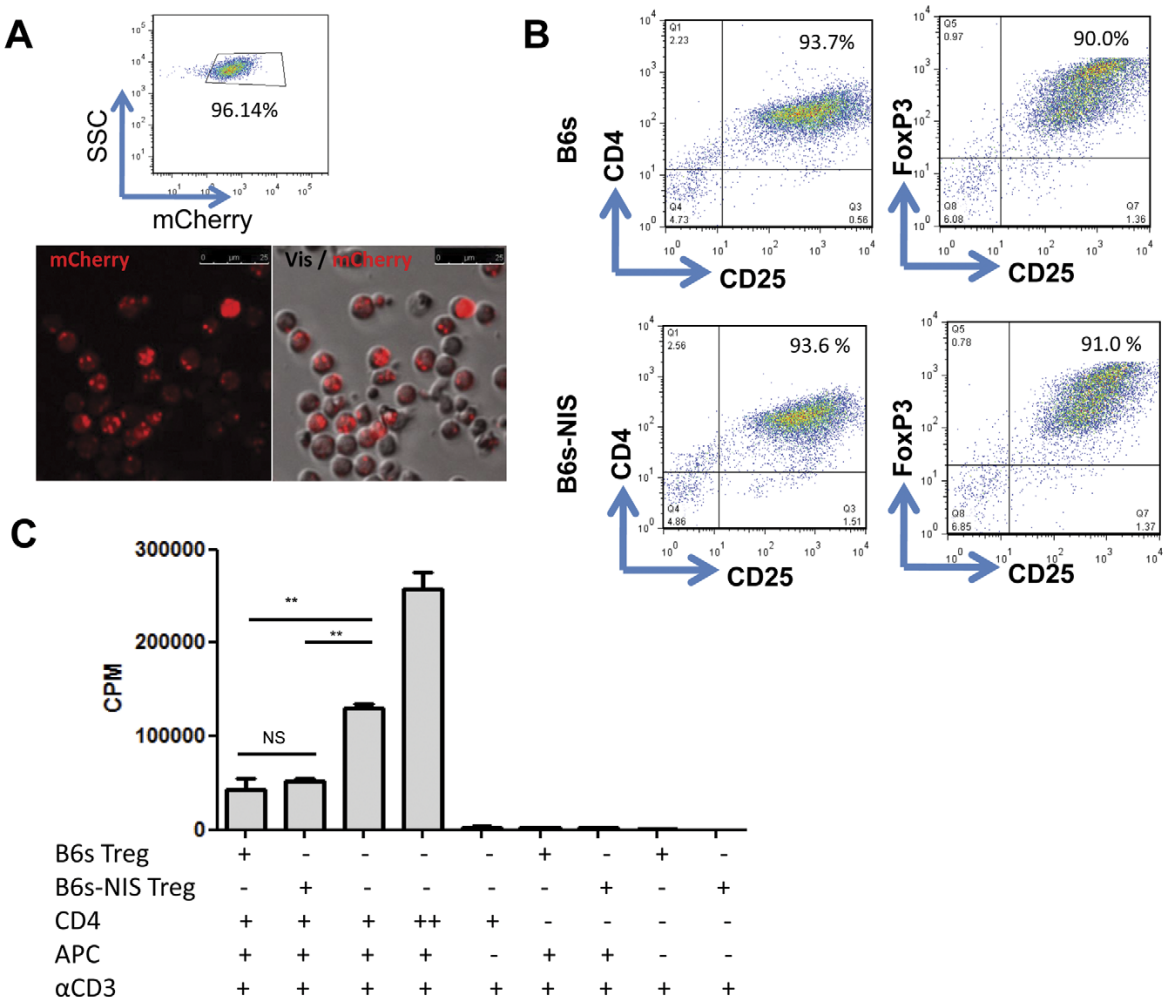
Tregs transduced with NIS maintained their phenotype and suppressive function *in vitro*. **A**) NIS transduced B6s Tregs were sorted by flow cytometry based on mCherry expression to 96.14%. NIS expressing Tregs co-express mCherry as shown using fluorescence microscopy. **B**) 5×10^5^ Tregs were stained for CD4-FITC, CD25-PE and FoxP3-APC. The result shown here are representative of 3 different experiments based on live cells using the forward and side scatter plots. **C**) Suppressive function of Treg. 5×10^4^ freshly isolated CD4^+^ T cells were cultured alone, or with transduced or non-transduced 5×10^4^ Tregs, in the presence of 1 µg/mL soluble purified anti- CD3ε and 1×10^5^ APC. T cell proliferation was measured by [^3^H]-thymidine incorporation (cpm) at 72 h of culture. Results are presented as mean cpm values of triplicate wells with standard error of the mean (SEM). **, P<0.003.

Previously we have demonstrated that self-specific Tregs generated from BL/6 mice stimulated with autologous DCs and IL-2 were suppressive *in vitro* and *in vivo* [19]. We have also shown that Tregs can be retrovirally transduced with myeloproliferative sarcoma virus to express an antigen-specific T cell receptor (TCR) whilst retaining their phenotypic and suppressive function [19]. In order to track adoptively transferred Tregs *in vivo* we employed a strategy by which we generated Tregs with defined antigen specificity and expressing the reporter gene NIS. Treg lines specific for self-MHC, generated from CD4^+^CD25^+^FoxpP3^+^ T cells, were retrovirally transduced with a DNA construct containing NIS and mCherry separated by a bicistronic IRES element. Although initially the transduction efficiency was low, with only 0.05% of Tregs expressing mCherry (data not shown), these cells (B6s-NIS Tregs) were expanded with autologous DCs and IL-2 and subsequently enriched for mCherry expression by flow cytometry cell sorting to achieve a population of cells that was 96% positive for mCherry expression (Fig. 1A). These B6s-NIS Tregs expressed similar levels of CD4, CD25 and FoxP3 compared to their non-transduced (B6s Tregs) counterparts (Fig. 1B) as well as maintaining their suppressive capacity (Fig. 1C). These observations suggested that retroviral administration of NIS and mCherry genes to CD4^+^CD25^+^FoxP3^+^ T cells and subsequent expansion did not alter their phenotype and suppressive function.

### NIS expressing Tregs can be radiolabelled with ^99m^TcO_4_^−^ in vitro

**Figure 2:**
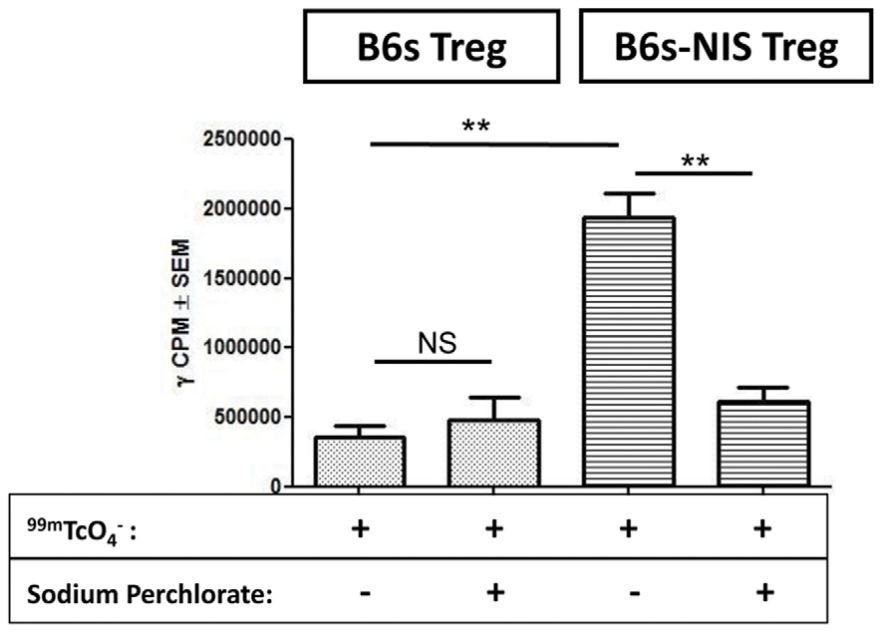
B6s-NIS Tregs uptake ^99m^TcO_4_^−^ *in vitro*. 1×10^6^ B6s or B6s-NIS Tregs were incubated in Hank's Balanced Salt solution supplemented with 1 MBq of ^99m^TcO_4_^−^. Sodium Perchlorate (100 µM) was used to block the ^99m^TcO_4_^−^ uptake by B6s-NIS Treg line. **, P<0.0082.

Previous studies have shown that cells such as rat glioma cells expressing NIS can be radiolabelled *in vitro* and *in vivo* with ^99m^TcO_4_^−^ [25]. As we plan to image NIS expressing Tregs *in vivo* using this radiolabel it was important to show that these cells express NIS and that the levels of NIS expressed was sufficient for mediated radiolabelling. NIS mediated uptake of ^99m^TcO_4_^−^ was assessed by incubating both the NIS expressing Tregs and the parental line with 1 MBq of ^99m^TcO_4_^−^ per 1×10^6^ cells. Incubation of NIS expressing Tregs with ^99m^TcO_4_^−^ resulted in accumulation of significant levels of this radiolabel into the cells (p = 0.0012) (Fig. 2). Moreover, further confirmation of the specificity of this receptor-mediated uptake was shown by addition of sodium perchlorate prior to radiolabelling. This chemical has previously been shown to inhibit the NIS receptor [18]. As expected, radiolabelling of ^99m^TcO_4_^−^ in the transduced cells was inhibited in the presence of sodium perchlorate (Fig. 2).

### Tregs radiolabelled with ^99m^TcO_4_^−^ maintained their viability, phenotype and suppressive function

**Figure 3:**
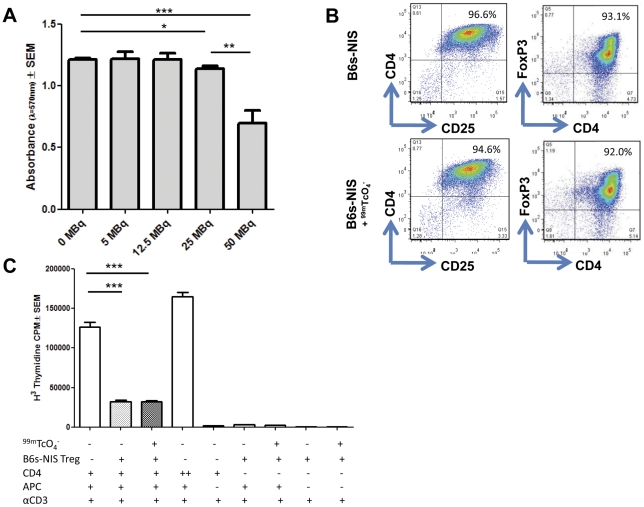
Effect of ^99m^TcO_4_^−^ uptake on the viability, phenotype and suppressive function of Tregs. **A**) Standard MTT assay was performed using 1×10^6^ B6s-NIS Tregs incubated in the presence of Hank's buffer supplemented with increasing ^99m^TcO_4_^−^ (ranging from 0–100 MBq of ^99m^TcO_4_^−^) in triplicates in a 96 well plate. **B**) B6s-NIS Tregs were incubated with or without ^99m^TcO_4_^−^ (30 MBq per million cells) and cultured *in vitro* for 7 days. These Tregs were then harvested for phenotypic and suppressive function. 5×10^5^ Tregs were stained with anti-CD4-FITC, anti-CD25-PE and anti-FoxP3-APC. Plots shown here are gated on live cells using the forward and side scatter plots. **C**) ^99m^TcO_4_^−^ labelling had no effect on suppressive function of B6s-NIS Tregs. 5×10^4^ freshly isolated CD4^+^ T cells were cultured alone, or with 5×10^4^ transduced Tregs, in the presence of 1 µg/mL soluble purified anti-CD3ε and 1×10^5^ APC. T cell proliferation was measured by [^3^H]-thymidine incorporation (cpm) at 72 h of culture. *, P<0.01; **, P<0.002; ***, P<0.0007.

As we wish to follow our Tregs longitudinally and throughout the lifespan of an immune response it was important to ensure that exposure to ^99m^TcO_4_^−^ did not affect the viability, phenotype or the function of NIS expressing Tregs. The NIS Tregs were exposed to increasing amounts of ^99m^TcO_4_^−^, ranging from 0–100 MBq per 1×10^6^ cells *in vitro*. Up to 25 MBq of ^99m^TcO_4_^−^ did not affect the viability of cells as measured by an MTT assay (Fig. 3A). As shown in Figure 3A cell death was seen only after incubation with 50 or 100 MBq (p = 0.0154 and p = 0.0006 respectively) of ^99m^TcO_4_^−^ as compared to unlabelled Tregs.

The effect of NIS mediated radiolabelling on the phenotype and suppressive function of Tregs was also analysed. In order to do so, NIS expressing Tregs were exposed to 30 MBq of ^99m^TcO_4_^−^ per million cells for fifteen minutes and both the phenotype of the cells as well as the suppressive function were studied 7 days later. ^99m^TcO_4_^−^ exposed B6s-NIS Tregs had similar surface levels of CD4, CD25 molecules and intracellular expression of FoxP3 as B6s-NIS Tregs not exposed to this radiolabel (Fig. 3B). In addition, no significant effect (p = 0.9296) on the suppressive capability of Tregs was observed following exposure to this radioisotope (Fig. 3C). From these observations we conclude that radiolabelling of B6s-NIS Tregs with ^99m^TcO_4_^−^ did not alter their viability, phenotype and suppressive capacity.

### NIS expressing Tregs can be imaged in vivo using NanoSPECT/CT

**Figure 4:**
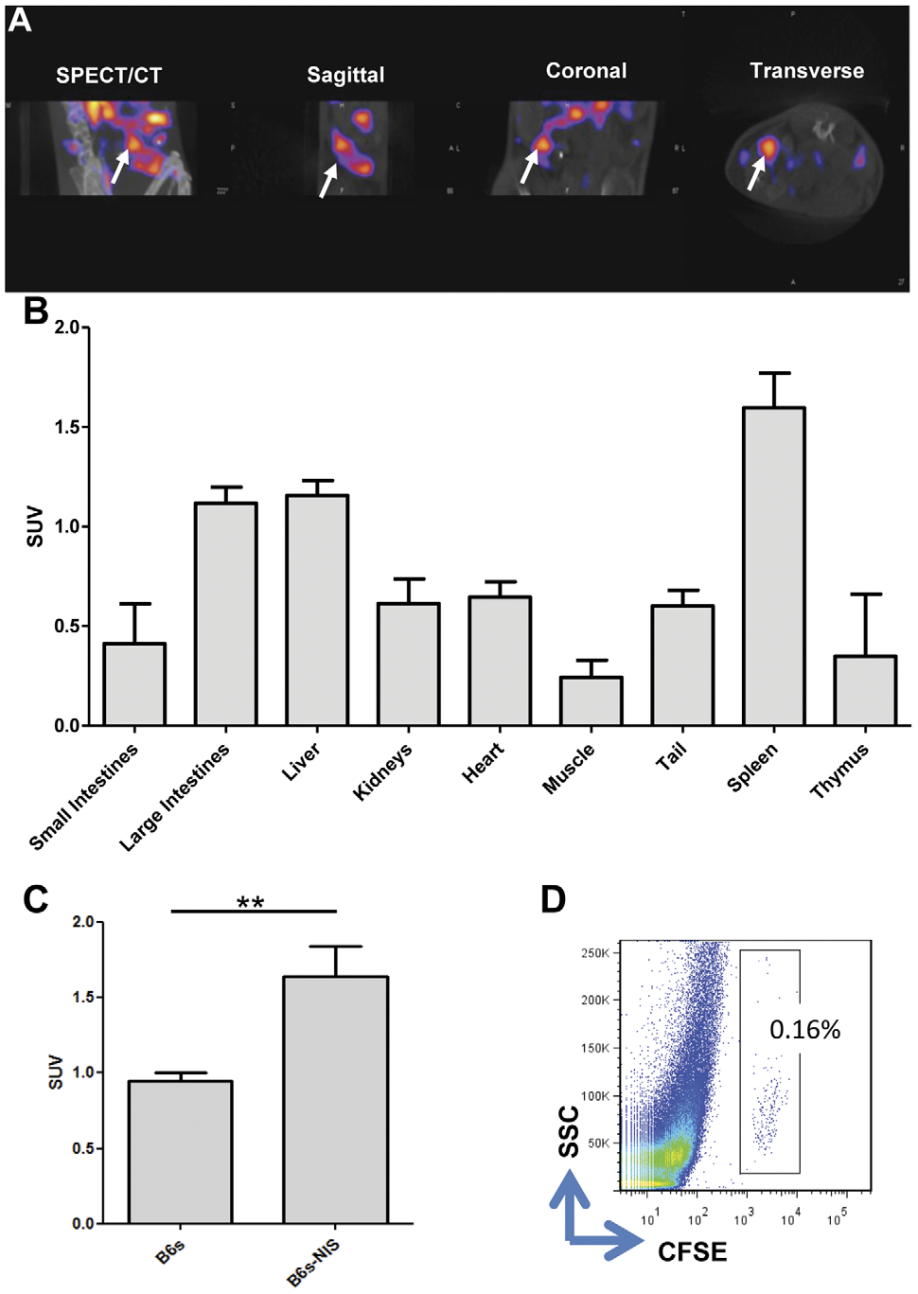
B6s-NIS Tregs can be imaged *in vivo* by NanoSPECT/CT through *in vivo* uptake of ^99m^TcO_4_^−^. **A**) BL/6 mice were adoptively transferred with 1×10^6^ CFSE labelled B6s-NIS Tregs 24 hours later mice received 20 MBq of ^99m^TcO_4_^−^ before being imaged using NanoSPECT/CT under general anaesthesia for 1 hour. The white arrow indicates the spleen in different views of scan. **B**) After scanning, mice were culled and vital organs were analysed for levels of ^99m^TcO_4_^−^. The graph illustrates the mean of Standard Uptake Value (SUV) of the organs (n = 3 mice). **C**) The SUV of spleens from mice adoptively transferred with B6s or B6s-NIS Tregs. Spleens containing B6s-NIS had significantly more ^99m^TcO_4_^−^ uptake (n = 6 per group). **D**) Flow Cytometry data showing the SSC and CFSE demonstrates the presence of CFSE labelled B6s-NIS Tregs in the spleen of injected mice. **, P<0.0082.

Next the *in vitro* observations with NIS expressing Tregs were extended *in vivo*. CFSE labelled NIS expressing Tregs were adoptively transferred into recipient BL/6 mice. One day later, mice were injected intravenously with ^99m^TcO_4_^−^ and imaged using NanoSPECT/CT. As expected, endogenously NIS expressing organs such as thyroid, stomach and salivary glands accumulated high levels of ^99m^TcO_4_^−^ (Fig. S1). Removal of these endogenously NIS signals using imaging software allowed the visualisation of other organs in which accumulated ^99m^TcO_4_^−^ was present. The NanoSPECT/CT image of B6s-NIS Tregs and ^99m^TcO_4_^−^ injected mice showed an accumulation of signal within the spleen (Fig. 4A). This was not observed in mice injected with non-transduced Tregs and ^99m^TcO_4_^−^ (Fig. S2). To further confirm these observations, mice were culled and ^99m^TcO_4_^−^ levels was measured in the vital organs (Fig. 4B). The results from the biodistribution analysis confirmed that the NIS expressing Tregs migrated to the spleen following adoptive transfer and they were radiolabelled *in vivo* with ^99m^TcO_4_^−^. When the levels of ^99m^TcO_4_^−^ in the spleens of animals receiving either B6s-NIS or B6s Tregs were compared a significant increase (p<0.0082) in radiolabel uptake was seen in the spleens of mice receiving NIS expressing Tregs (Fig. 4C). Lastly, we confirmed that the radioactive signals seen in the spleens, both in the scan and in the organ biodistribution, were due to the presence of NIS Tregs cells as we looked for the presence of CFSE labelled Tregs within this organ via flow cytometry. Indeed, CFSE positive Tregs were present in the spleens of mice receiving NIS or untransduced Tregs (Fig. 4D) confirming that the differences in ^99m^TcO_4_^−^ levels noticed was due to NIS expressing cells residing in the spleen rather than passive uptake of this radioisotope.

We concluded that Tregs expressing NIS can be radiolabelled with ^99m^TcO_4_^−^ *in vitro* and more importantly *in vivo* as shown by NanoSPECT/CT. Furthermore, we observed that adoptively transferred Tregs were located predominantly within the spleen of recipient mice 24 hours after administration.

## Discussion

It is well established that Tregs have an important role in down-regulating the immune response. It is this suppressive function of Tregs that has made them a potential tool for adoptive cell transfer and therapy for immune related diseases such as autoimmune diseases and transplantation. Various imaging based studies have helped in dissecting some of the mechanisms of action of Tregs [26] these include: 1) the importance of Treg-DC interaction for Treg function [27], 2) the relevance of sequential migration of Tregs from blood to graft and then to the draining lymph node for graft survival [8] and 3) during a skin immune response in the form of contact hypersensitivity [9]. However, there are some limitations associated with the imaging techniques used in the reported studies including the difficulty of their translation to the clinical studies [28]. PET and SPECT are examples of imaging techniques that are used in the nuclear medicine and allows tracking and monitoring of cells in the whole body for long periods of time [13]. Immune cells have been so far directly radiolabelled for imaging using PET and SPECT, and the major disadvantage of this approach is that they can be visualised for very short period depending upon the half-life of the radioisotope used [13]. Radiolabelling cells through NIS, used in our study, allows receptor mediated uptake of specific radioisotopes such as ^99m^TcO_4_^−^ for as long as the cells are alive. Tumour cell lines expressing NIS have been generated and adoptively transferred into animals to study *in vivo* cancer development and tumour therapy [18]. These studies suggest that receptor mediated radiolabelling can be utilised to image adoptively transferred cells. However we are the first group to utilise NIS mediated labelling to track immune cells and in particular Tregs. We have demonstrated that Tregs can be retrovirally transduced to express NIS without any alteration to their phenotype and function *in vitro*. B6s-NIS Tregs were enriched by cell sorting based on the expression of mCherry, as previously reported [29]. We then demonstrated that NIS expressing Tregs can be radiolabelled *in vitro* and more importantly *in vivo* with ^99m^TcO_4_^−^ and that this radiolabelling is NIS dependent. In addition, exposure to less than 30 MBq of ^99m^TcO_4_^−^ per 1×10^6^ of cells did not alter the phenotype or the suppressive function of B6s-NIS Tregs. Therefore, for *in vivo* uptake and imaging studies, 30 or less MBq of ^99m^TcO_4_^−^ was utilised. Finally we observed that NIS expressing Tregs are located within the spleen of recipient mice 24 hours following adoptive transfer and injection of ^99m^TcO_4_^−^ in mice using NanoSPECT/CT as previously demonstrated with adoptively transferred T cells using SPECT [30] Bioluminescence and PET Imaging [31].

Adoptive transfer of NIS transduced Tregs will allow the expansion of our knowledge regarding the life span, localization, distribution and migration patterns of these cells in various models. Furthermore, the influence of antigen-specificity for Treg localisation and function can be investigated, having demonstrated that murine and human antigen-specific Tregs are superior comparing to polyclonal Tregs in protecting from heart and skin transplant rejections [5], [19]. Moreover, Tregs have been shown to be heterogeneous in phenotype and function [32]. The PET and SPECT imaging technology could help in clarifying the mechanism of action and migration patterns of Treg subsets *in vivo*. Finally this technology is a pre-clinical imaging technique making it applicable for clinical studies of adoptive Treg therapy to further refine future strategies to induce tolerance in the context of transplantation and autoimmune diseases.

## Supporting Information

Figure S1 **B6s-NIS Tregs can be imaged** ***in vivo*** **using NanoSPECT/CT.** BL/6 mice were adoptively transferred with 1×10^6^ CFSE labelled B6s-NIS Tregs 24 hours later mice received 20 MBq of ^99m^TcO_4_^−^ before being imaged using NanoSPECT/CT under general anaesthesia for 1 hour. **A**) SPECT/CT Image acquired showing Stomach, Salivary glands and Thyroid. **B**) Using the InvivoScope software, the signals from Stomach was removed allowing the visualisation of the spleen.
(AVI)

Figure S2 **B6s Tregs cannot be imaged** ***in vivo*** **using NanoSPECT/CT.** BL/6 mice were adoptively transferred with 1×10^6^ CFSE labelled B6s Tregs 24 hours later mice received 20 MBq of ^99m^TcO_4_^−^ before being imaged using NanoSPECT/CT under general anaesthesia for 1 hour. **A**) SPECT/CT Image acquired showing Stomach, Salivary glands and Thyroid. **B**) Using the InvivoScope software, the signals from Stomach was removed. **C**) After scanning, mice were culled and vital organs were analysed for levels of ^99m^TcO_4_^−^. The graph illustrates the mean of Standard Uptake Value (SUV) of the organs (n = 3 mice).
(AVI)
